# Supplementary material for: Specific GPCRs Elicit Unique Extracellular Vesicle MiRNA Array Signatures: An Exploratory Study
Source: bioRxiv. 2025 Nov 21:2025.06.16.659918. Originally published 2025 Jun 20. Preprint. [Version 2] doi: 10.1101/2025.06.16.659918 (PMC12262688; doi:10.1101/2025.06.16.659918)

bioRxiv preprint doi: https://doi.org/10.1101/2025.06.16.659918; this version posted November 21, 2025. The copyright holder for this preprint (which was not certified by peer review) is the author/funder. This article is a US Government work. It is not subject to copyright under 17 USC 105 and is also made available for use under a CC0 license.

Supplemental Materials

Figure S1. Sensitivity analysis across alternative estimates of treatment effects. (**A**) Scatterplot matrix showing pairwise concordance of alternative treatment-effect estimators, including all per-miRNA estimates from all receptors combined, with text labels (in quotes) indexed to the table below; note that the perfect correlation in the "individual" and "matched" approaches is expected, and the "pooled" and "batched" approaches only apply to the receptors ADORA1 and H1R. All estimators not based on matched pairs demonstrate bias in the form of exaggeration of effect size on average, and estimators employing a higher degree of aggregation (either at the treatment group or batch level) show worse correlation with the unbiased matched-pairs estimator (an example of ecological bias); it is clear that process variance brings considerable risk of bias in experiments of this type, and that great care should be taken to provide adequate representation of process variance via controls across experiments. (**B**) Comparison table detailing the differences between alternative estimators, including advantages and disadvantages of each approach. Our preferred approach is "matched", on both theoretical and empirical grounds. Briefly, the six combinations were: 1) treatment effect estimated as difference of group medians with permutation-based p-value; 2) difference of group medians with p-value from Welch's robust t-test; 3) (applies to batched receptors only) treatment coefficient from median regression of pooled-batch data with p-value from the rank-based Mann-Whitney test; 4) (applies to batched receptors only) sample-weighted average of treatment coefficients from separate median regressions of data from each batch with p-value as the harmonic mean of Mann-Whitney p-values calculated separately on each batch; 5) mean matched-pairs estimator (simple average of all within-pair differences between agonist and vehicle control) with permutation-based p-value; and 6) regression matched-pairs estimator (treatment coefficient from a within-pairs linear regression) with p-value from the unbalanced-robust rank-based Skillings-Mack test. Note that (1) was the only approach where we applied group-based normalization (for all others we used run-based), and that the treatment effects in (5) and (6) are not strictly the same except in the absence of missing data but they are operationally 100% correlated.

bioRxiv preprint doi: https://doi.org/10.1101/2025.06.16.659918; this version posted November 21, 2025. The copyright holder for this preprint (which was not certified by peer review) is the author/funder. This article is a US Government work. It is not subject to copyright under 17 USC 105 and is also made available for use under a CC0 license.

Table S1. The analysis of GPCR gene expression across various cell lines, as sourced from the Human Protein Atlas.

Figure S2. Representative immunoblots showed stimulation of ACKR3 with SDF-1α-induced phosphorylation of ERK1/2 (pERK1/2) compared to total ERK1/2 (tERK1/2). β-actin was used as a loading control.

Figure S3. Quantification of EVs in U2OS media in response to receptor activation. U2OS cells were incubated with vehicle control (VC) or a selective agonist CCPA (**A**), PEA (**B**), Wnt3a (**C**), or SDF-1α (**D**) for ADORA1, HRH1, FZD4, and ACKR3, respectively. EVs were isolated and the concentration was normalized as a percentage of the media control (MC). EV size distribution following agonist or VC treatment in ADORA1 (**E**), HRH1 (**F**), FZD4 (**G**), and ACKR3 (**H**). Data are shown as mean ± SEM, n=3. A t-test determined no significant differences in EV concentration or size distribution between the VC and agonist stimulation in all GPCRs, $p > 0.05$.

Table S2. miRNAs detected in media control.

Table S3. Differentially expressed miRNAs in the isolated EVs following GPCR activation. All differentially expressed miRNAs (meeting $p < 0.2$) after stimulation were listed.

Figure S4. The top 10 hub genes identified in miRNA targets (≥ 1.5-fold change) PPI network.

Figure S5. Pathway analysis of miR-502-3p and miR-137 targets.

The bubble plots showed the top 25 significantly enriched KEGG pathways for the targets of (**A**) miR-502-3p and (**B**) miR-137. The dot size represents the number of enriched gene targets, and the color shows the *p*-value of the enrichment. For the enrichment analysis, the cut-off criteria were *p*-value (FDR) < 0.05 and gene count > 2. KEGG term abbreviations: ** Aldosterone-regulated sodium reabsorption.

bioRxiv preprint doi: https://doi.org/10.1101/2025.06.16.659918; this version posted November 21, 2025. The copyright holder for this preprint (which was not certified by peer review) is the author/funder. This article is a US Government work. It is not subject to copyright under 17 USC 105 and is also made available for use under a CC0 license.

SUPPLEMENTARY FIGURES AND TABLES

Figure S1

A

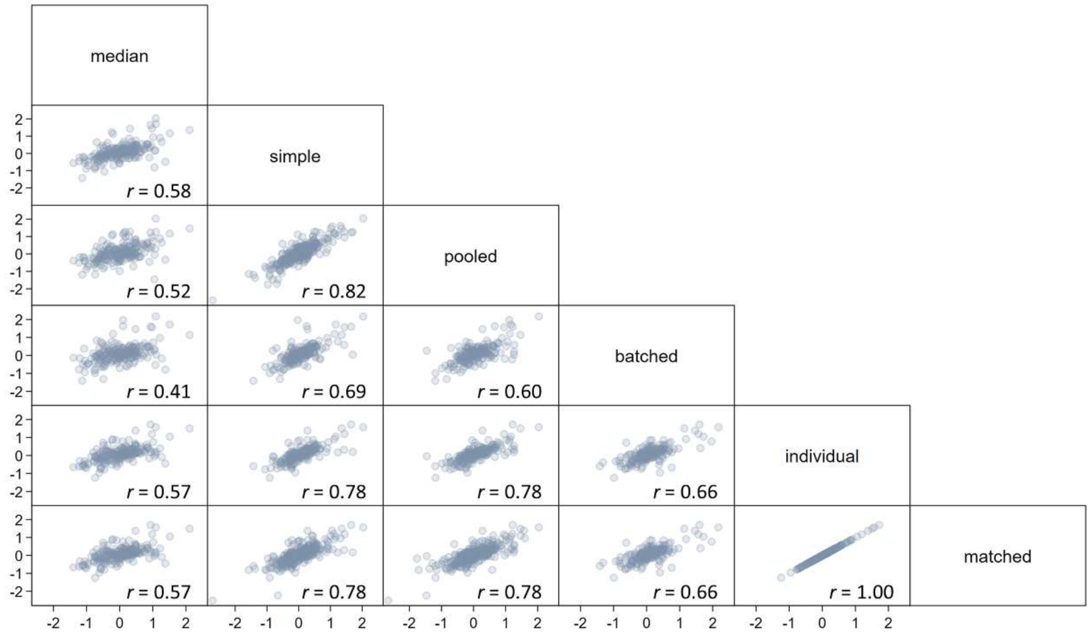

B

Method	Normalization	Effect estimate	P-value	PROS	CONS
Group medians (original = "median")	Align group medians	$Median_1 - Median_0$	Permutation test	Easy to do	Retains within-group technical variance
Group medians (new = "simple")	Align run medians	$Median_1 - Median_0$	Robust t-test	Easy to do	P-value sensitive to outlier observations
Pooled-batches ("pooled")	Align run medians	β_{TX} coefficient from median regression	Mann-Whitney test	Accurate p-value	Ignores batch differences in treatment effects
Batch-averaged ("batched")	Align run medians	Weighted average of the β_{TX1} and β_{TX2} coefficients from batch-specific median regressions	Harmonic mean of the p-values from batch-specific Mann-Whitney tests	Accounts for batch differences in treatment effects	Low power, sensitive to batch differences in missingness
Matched-pairs (mean = "individual")	Align run medians	Simple average of all pair-specific TX – VC differences	Permutation test	Accounts for any correlation of process order with treatment effects (stronger than a batch correction!)	P-value sensitive to process order of missing pairs
Matched-pairs (regression = "matched")	Align run medians	β_{TX} coefficient from within-pairs (aka FE) linear regression	Skillings-Mack test	Accurate p-value	NONE

bioRxiv preprint doi: https://doi.org/10.1101/2025.06.16.659918; this version posted November 21, 2025. The copyright holder for this preprint (which was not certified by peer review) is the author/funder. This article is a US Government work. It is not subject to copyright under 17 USC 105 and is also made available for use under a CC0 license.

Table S1

Cell line	Gene	Gene description	UniProt evidence
SH-SY5Y	GRM7	Glutamate metabotropic receptor 7	Evidence at protein level
	ADCYAP1R1	ADCYAP receptor type I	Evidence at protein level
	ADGRA2	Adhesion G protein-coupled receptor A2	Evidence at protein level
	ADGRL2	Adhesion G protein-coupled receptor L2	Evidence at protein level
	PTGDR	Prostaglandin D2 receptor	Evidence at transcript level
	VN1R1	Vomeronasal 1 receptor 1	Evidence at transcript level
A-431	ADGRG2	Adhesion G protein-coupled receptor G2	Evidence at protein level
	ADORA2B	Adenosine A2b receptor	Evidence at protein level
	ADRB2	Adrenoceptor beta 2	Evidence at protein level
	CELSR2	Cadherin EGF LAG seven-pass G-type receptor 2	Evidence at protein level
	EDNRB	Endothelin receptor type B	Evidence at protein level
	FZD3	Frizzled class receptor 3	Evidence at protein level
	FZD6	Frizzled class receptor 6	Evidence at protein level
	GPR87	G protein-coupled receptor 87	Evidence at transcript level
	GRM1	Glutamate metabotropic receptor 1	Evidence at protein level
	OR10Q1	Olfactory receptor family 10 subfamily Q member 1	Evidence at transcript level
	P2RY2	Purinergic receptor P2Y2	Evidence at protein level
	SSTR2	Somatostatin receptor 2	Evidence at protein level
	TACR2	Tachykinin receptor 2	Evidence at protein level
U-251MG	HTR7	5-hydroxytryptamine receptor 7	Evidence at protein level
	S1PR1	Sphingosine-1-phosphate receptor 1	Evidence at protein level
	ADRA1B	Adrenoceptor alpha 1B	Evidence at protein level
	CCR4	C-C motif chemokine receptor 4	Evidence at protein level
	EDNRA	Endothelin receptor type A	Evidence at protein level
	FZD1	Frizzled class receptor 1	Evidence at protein level
	GPR142	G protein-coupled receptor 142	Evidence at transcript level
	GPRC5B	G protein-coupled receptor class C group 5 member B	Evidence at transcript level
	LGR6	Leucine rich repeat containing G protein-coupled receptor 6	Evidence at protein level
	MAS1L	MAS1 proto-oncogene like, G protein-coupled receptor	Evidence at transcript level
	OR6P1	Olfactory receptor family 6 subfamily P member 1	No human protein/transcript evidence
	PTAFR	Platelet activating factor receptor	Evidence at protein level
U2OS	GPRC5A	G protein-coupled receptor class C group 5 member A	Evidence at protein level
	GLP2R	Glucagon like peptide 2 receptor	Evidence at protein level
	NMBR	Neuromedin B receptor	Evidence at protein level
	OR4N4	Olfactory receptor family 4 subfamily N member 4	Evidence at transcript level
	ACKR2	Atypical chemokine receptor 2	Evidence at protein level
	ACKR3	Atypical chemokine receptor 3	Evidence at protein level
	ADGRE5	Adhesion G protein-coupled receptor E5	Evidence at protein level
	ADGRF4	Adhesion G protein-coupled receptor F4	Evidence at protein level
	ADORA1	Adenosine A1 receptor	Evidence at protein level
	CALCRL	Calcitonin receptor like receptor	Evidence at protein level

bioRxiv preprint doi: https://doi.org/10.1101/2025.06.16.659918; this version posted November 21, 2025. The copyright holder for this preprint (which was not certified by peer review) is the author/funder. This article is a US Government work. It is not subject to copyright under 17 USC 105 and is also made available for use under a CC0 license.

	CCR7	C-C motif chemokine receptor 7	Evidence at protein level
	CRHR2	Corticotropin releasing hormone receptor 2	Evidence at protein level
	DRD4	Dopamine receptor D4	Evidence at protein level
	F2RL1	F2R like trypsin receptor 1	Evidence at protein level
	FZD4	Frizzled class receptor 4	Evidence at protein level
	FZD8	Frizzled class receptor 8	Evidence at protein level
	GABBR1	Gamma-aminobutyric acid type B receptor subunit 1	Evidence at protein level
	GCGR	Glucagon receptor	Evidence at protein level
	GPR156	G protein-coupled receptor 156	Evidence at transcript level
	GPR162	G protein-coupled receptor 162	Evidence at protein level
	GPR17	G protein-coupled receptor 17	Evidence at protein level
	GPR173	G protein-coupled receptor 173	Evidence at protein level
	GPR27	G protein-coupled receptor 27	Evidence at transcript level
	GPR34	G protein-coupled receptor 34	Evidence at transcript level
	GPR63	G protein-coupled receptor 63	Evidence at transcript level
	HRH1	Histamine receptor H1	Evidence at protein level
	NPFFR2	Neuropeptide FF receptor 2	Evidence at protein level
	S1PR4	Sphingosine-1-phosphate receptor 4	Evidence at protein level
	SSTR3	Somatostatin receptor 3	Evidence at protein level
	VIPR2	Vasoactive intestinal peptide receptor 2	Evidence at protein level

bioRxiv preprint doi: https://doi.org/10.1101/2025.06.16.659918; this version posted November 21, 2025. The copyright holder for this preprint (which was not certified by peer review) is the author/funder. This article is a US Government work. It is not subject to copyright under 17 USC 105 and is also made available for use under a CC0 license.

Figure S2

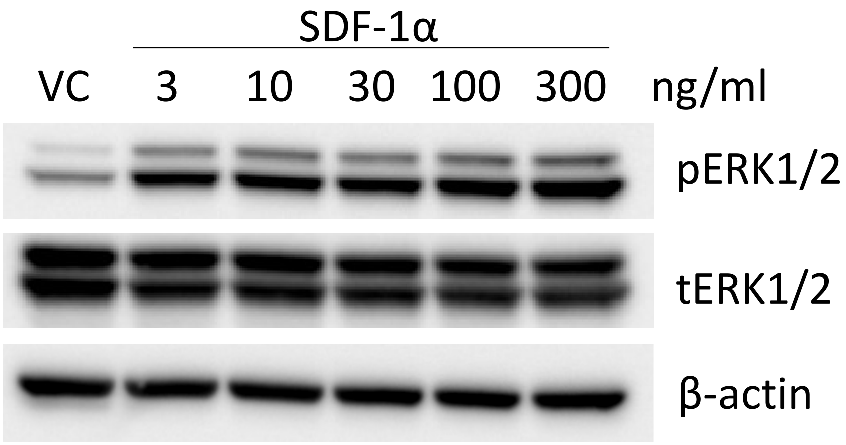

bioRxiv preprint doi: https://doi.org/10.1101/2025.06.16.659918; this version posted November 21, 2025. The copyright holder for this preprint (which was not certified by peer review) is the author/funder. This article is a US Government work. It is not subject to copyright under 17 USC 105 and is also made available for use under a CC0 license.

Figure S3

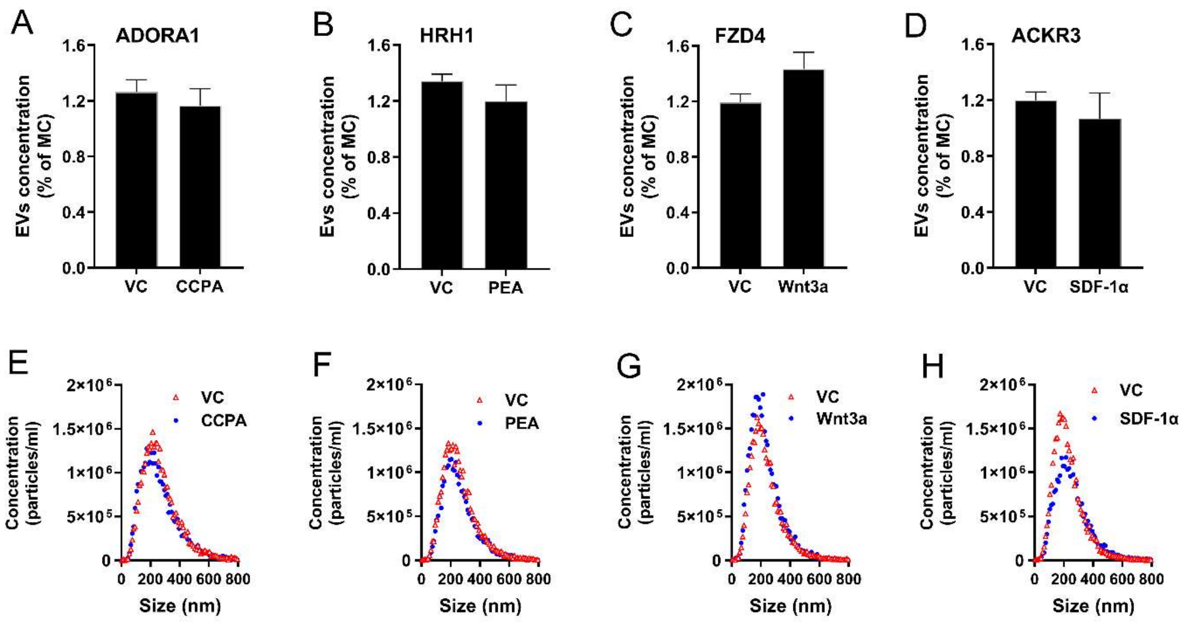

bioRxiv preprint doi: https://doi.org/10.1101/2025.06.16.659918; this version posted November 21, 2025. The copyright holder for this preprint (which was not certified by peer review) is the author/funder. This article is a US Government work. It is not subject to copyright under 17 USC 105 and is also made available for use under a CC0 license.

Table S2

miRNA	Average Ct	SD
hsa-miR-106b-5p	25.7	1.4
hsa-miR-126-5p	27.0	0.8
hsa-miR-126-3p	24.6	1.1
hsa-miR-127-3p	26.4	0.7
hsa-miR-1305	30.4	0.2
hsa-miR-142-3p	26.4	1.6
hsa-miR-150-5p	26.4	0.6
hsa-miR-15b-5p	27.8	1.0
hsa-miR-192-5p	28.5	0.8
hsa-miR-194-5p	29.6	0.3
hsa-miR-204-5p	28.8	0.5
hsa-miR-215-5p	29.0	0.6
hsa-miR-223-3p	23.1	0.9
hsa-miR-25-3p	28.0	0.8
hsa-miR-26a-5p	26.1	0.8
hsa-miR-26b-5p	29.1	1.0
hsa-miR-27b-3p	30.1	0.2
hsa-miR-30b-5p	25.0	0.8
hsa-miR-30c-5p	25.0	1.2
hsa-miR-331-3p	25.6	0.8
hsa-miR-338-5p	24.5	0.1
hsa-miR-376a-3p	28.7	0.5
hsa-miR-378	28.8	2.6
hsa-miR-409-3p	26.9	1.3
hsa-miR-494-3p	28.5	0.9
hsa-miR-505-5p	32.3	0.8
hsa-miR-520c-3p	26.3	0.2
hsa-miR-520d-3p	25.8	0.9
hsa-miR-539-5p	29.2	1.2
hsa-miR-548a-3p	30.6	0.4
hsa-miR-572	26.7	0.3
hsa-miR-601	29.2	0.6
hsa-miR-625-3p	28.1	0.2
hsa-miR-628-5p	31.1	0.6
hsa-miR-875-5p	32.3	0.5
hsa-miR-451a	24.3	1.2

bioRxiv preprint doi: https://doi.org/10.1101/2025.06.16.659918; this version posted November 21, 2025. The copyright holder for this preprint (which was not certified by peer review) is the author/funder. This article is a US Government work. It is not subject to copyright under 17 USC 105 and is also made available for use under a CC0 license.

Table S3

**miRNA**	**Log_2 fold change**	***p* value**
**Adenosine A1 receptor (ADORA1)**		
miR-550a-5p	1.7	0.0455
miR-1227-3p	1.49	0.0253
miR-454-5p	1.17	0.0143
miR-340-3p	0.85	0.1797
miR-95-3p	0.73	0.1024
miR-31-5p	0.58	0.0143
miR-592	0.55	0.1797
miR-100-5p	0.42	0.0253
let-7d-5p	0.39	0.1024
miR-99a-5p	0.36	0.0253
miR-454-3p	0.35	0.1024
miR-99b-5p	0.31	0.1797
miR-708-5p	0.28	0.1024
miR-1291	0.16	0.1797
miR-92a-3p	0.11	0.1024
miR-186-5p	0.1	0.0143
miR-106a-5p	0.1	0.1797
miR-135b-5p	0.09	0.1024
miR-27a-3p	-0.22	0.1024
miR-339-3p	-0.29	0.1024
miR-942-5p	-0.32	0.1024
miR-148a-3p	-0.33	0.1024
miR-577	-0.33	0.1797
miR-130a-3p	-0.46	0.1024
miR-769-5p	-0.52	0.1024
miR-361-5p	-0.62	0.1797
miR-1180-3p	-0.66	0.1797
miR-660-5p	-0.74	0.1024
miR-135a-5p	-0.79	0.0143
**Histamine receptor H1 (HRH1)**		
miR-502-3p	1.57	0.0253
miR-423-5p	0.59	0.0253
miR-34a-5p	0.57	0.1797
miR-365a-3p	0.54	0.1024
miR-519a-3p	0.38	0.1797
miR-199a-3p	0.37	0.0143
miR-130a-3p	0.35	0.1024
miR-99b-5p	0.34	0.1024
miR-708-5p	0.25	0.1024
miR-20b-5p	0.18	0.1797
miR-222-3p	0.16	0.1797
miR-197-3p	-0.11	0.1024

bioRxiv preprint doi: https://doi.org/10.1101/2025.06.16.659918; this version posted November 21, 2025. The copyright holder for this preprint (which was not certified by peer review) is the author/funder. This article is a US Government work. It is not subject to copyright under 17 USC 105 and is also made available for use under a CC0 license.

miR-181a-2-3p	-0.18	0.1024
miR-146a-5p	-0.21	0.1024
miR-146b-5p	-0.23	0.1024
miR-139-3p	-0.28	0.0455
miR-483-5p	-0.35	0.1024
miR-214-5p	-0.38	0.1024
miR-345-5p	-0.38	0.1024
miR-1180-3p	-0.42	0.1024
miR-339-5p	-0.54	0.1797
miR-744-5p	-0.62	0.0253
miR-1291	-1.25	0.1024
**Frizzled class receptor 4 (FZD4)**		
miR-518e-3p	1.39	0.0833
miR-422a	1.2	0.1024
miR-564	1.1	0.0833
miR-520e-3p	0.96	0.0833
miR-320b	0.78	0.1024
miR-135b-3p	0.72	0.1797
miR-375-3p	0.48	0.1024
miR-181a-3p	0.41	0.1797
miR-577	0.34	0.0143
miR-1227-3p	0.33	0.1797
miR-15b-3p	0.15	0.1024
miR-455-5p	0.15	0.1024
miR-28-3p	-0.09	0.1024
miR-483-5p	-0.46	0.1024
miR-95-3p	-0.5	0.0143
miR-590-3p	-0.5	0.1024
miR-660-5p	-0.51	0.0143
miR-625-5p	-0.51	0.1797
miR-532-5p	-0.69	0.1024
miR-203a-3p	-0.9	0.0143
miR-190a-5p	-2.24	0.0833
miR-137-3p	-2.52	0.1797
**Atypical chemokine receptor 3 (ACKR3)**		
miR-422a	1.11	0.1797
miR-1255b-5p	0.7	0.1797
miR-135b-3p	0.67	0.0253
miR-10b-5p	0.63	0.1797
miR-516a-3p	0.55	0.1797
miR-1227-3p	0.49	0.1024
miR-9-3p	0.37	0.0143
miR-9-5p	0.32	0.1024
miR-324-5p	0.3	0.0143
miR-26a-1-3p	0.29	0.1024
miR-152-3p	0.28	0.0143
miR-221-3p	0.26	0.1797
miR-31-3p	0.26	0.1024

bioRxiv preprint doi: https://doi.org/10.1101/2025.06.16.659918; this version posted November 21, 2025. The copyright holder for this preprint (which was not certified by peer review) is the author/funder. This article is a US Government work. It is not subject to copyright under 17 USC 105 and is also made available for use under a CC0 license.

miR-1180-3p	0.21	0.0833
miR-708-5p	0.19	0.1024
miR-125b-1-3p	0.16	0.1797
miR-590-5p	0.14	0.1024
miR-339-3p	-0.17	0.0143
miR-191-3p	-0.23	0.1024
miR-125a-5p	-0.3	0.0143
miR-502-3p	-0.6	0.1797
miR-642a-5p	-0.7	0.1024

bioRxiv preprint doi: https://doi.org/10.1101/2025.06.16.659918; this version posted November 21, 2025. The copyright holder for this preprint (which was not certified by peer review) is the author/funder. This article is a US Government work. It is not subject to copyright under 17 USC 105 and is also made available for use under a CC0 license.

Figure S4

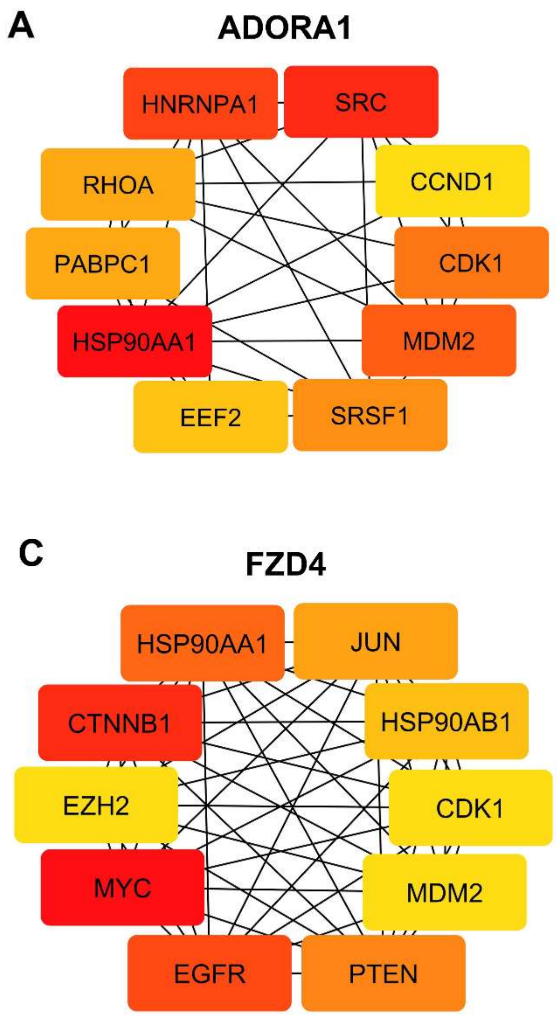

bioRxiv preprint doi: https://doi.org/10.1101/2025.06.16.659918; this version posted November 21, 2025. The copyright holder for this preprint (which was not certified by peer review) is the author/funder. This article is a US Government work. It is not subject to copyright under 17 USC 105 and is also made available for use under a CC0 license.

Figure S5

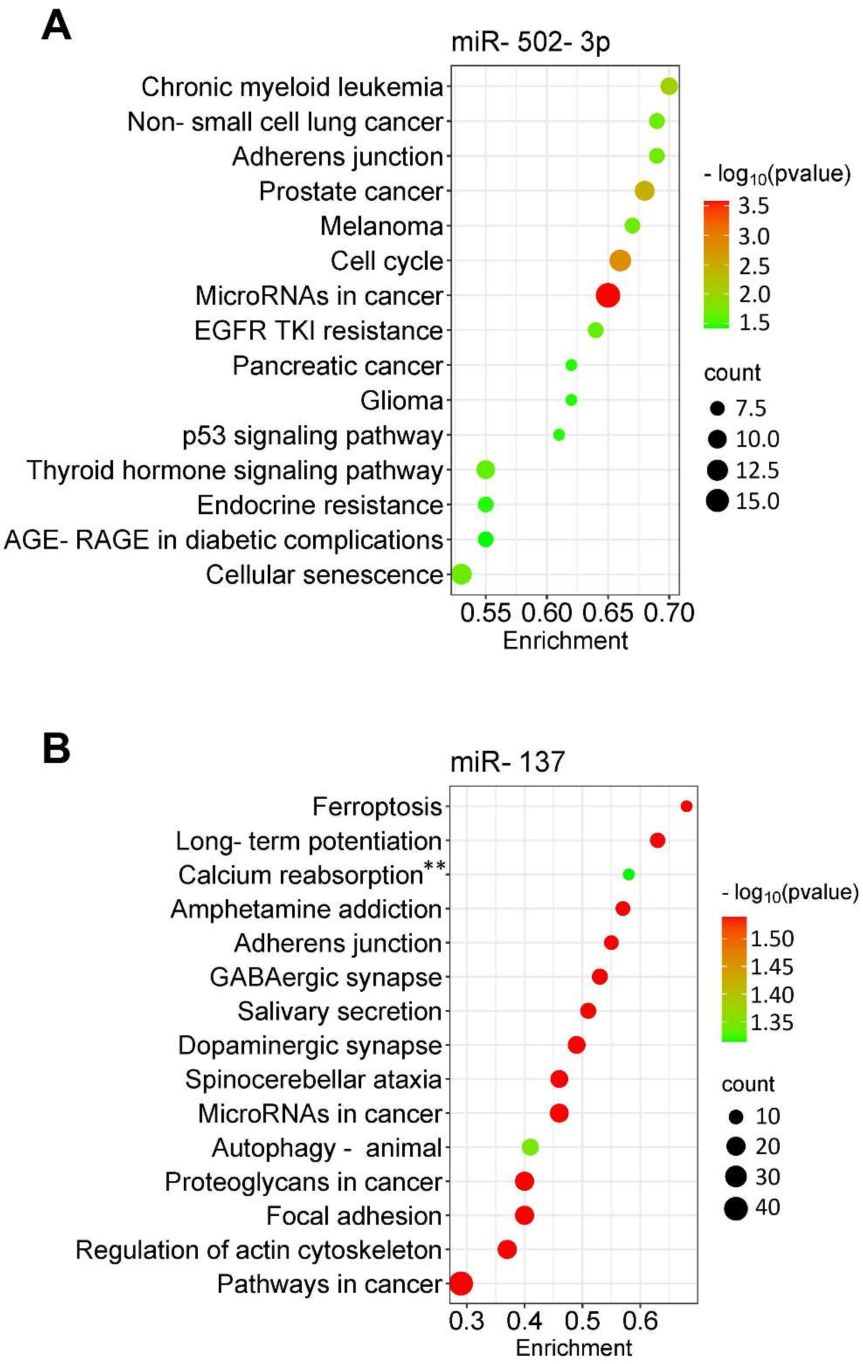

Supplement: Supplement 1 [file NIHPP2025.06.16.659918v2-supplement-1.pdf]
